# Characteristics of the paravertebral muscle in adult degenerative scoliosis with PI-LL match or mismatch and risk factors for PI-LL mismatch

**DOI:** 10.3389/fsurg.2023.1111024

**Published:** 2023-03-29

**Authors:** Jichao Guo, Dongxiao Xie, Jinniu Zhang, Wenyuan Ding, Boyang Zhao, Zhaohui Li, Yachong Huo

**Affiliations:** ^1^ Department of Orthopedic, Third Hospital of Hebei Medical University, Shijiazhuang, China; ^2^ Department of Pediatric Orthopedic, Third Hospital of Hebei Medical University, Shijiazhuang, China; ^3^ Department of Psychiatry, First Hospital of Hebei Medical University, Shijiazhuang, China; ^4^ Department of Spine Surgery, Third Hospital of Hebei Medical University, Shijiazhuang, China

**Keywords:** adult degenerative scoliosis, paravertebral muscle, fat infiltration area, PI-LL mismatch, risk factor

## Abstract

**Objective:**

Pelvic incidence (PI) minus the lumbar lordosis (LL) angle (PI-LL) correlates with function and disability. It is associated with paravertebral muscle (PVM) degeneration and is a valuable tool for surgical planning of adult degenerative scoliosis (ADS). This study aims to explore the characteristics of PVM in ADS with PI-LL match or mismatch and to identify the risk factors for PI-LL mismatch.

**Methods:**

A total of 67 patients with ADS were divided into PI-LL match and mismatch groups. The visual analog scale (VAS), symptom duration, and Oswestry disability index (ODI) were used to assess patients’ clinical symptoms and quality of life. The percentage of fat infiltration area (FIA%) of the multifidus muscle at the L1-S1 disc level was measured by using MRI with Image-J software. Sagittal vertical axis, LL, pelvic tilt (PT), PI, sacral slope, and the asymmetric and average degeneration degree of the multifidus were recorded. Logistic regression analysis was done to identify the risk factors for PI-LL mismatch.

**Results:**

In the PI-LL match and mismatch groups, the average FIA% of the multifidus on the convex side was less than that on the concave side (*P* < 0.05). There was no statistical difference of asymmetric degeneration degree of the multifidus between the two groups (*P* > 0.05). In the PI-LL mismatch group, the average degeneration degree of the multifidus, VAS, symptom duration, and ODI were significantly higher than that in the PI-LL match group, respectively (32.22 ± 6.98 vs. 26.28 ± 6.23 (%), 4.33 ± 1.60 vs. 3.52 ± 1.46, 10.81 ± 4.83 vs. 6.58 ± 4.23 (month), 21.06 ± 12.58 vs. 12.97 ± 6.49, *P* < 0.05). The average degeneration degree of the multifidus muscle was positively correlated with the VAS, symptom duration, and ODI, respectively (*r* = 0.515, 0.614, and 0.548, *P* < 0.05). Sagittal plane balance, LL, PT, and the average degeneration degree of the multifidus were the risk factors for PI-LL mismatch (OR: 15.447, 95% CI: 1.274–187.269; OR: 0.001, 95% CI: 0.000–0.099; OR: 107.540, 95% CI: 5.195–2,225.975; OR: 52.531, 95% CI: 1.797–1,535.551, *P* < 0.05).

**Conclusion:**

The PVM on the concave side was larger than that on the convex side in ADS irrespective of whether PI-LL matched or not. PI-LL mismatch could aggravate this abnormal change, which is an important cause of pain and disability in ADS. Sagittal plane imbalance, decreased LL, higher PT, and larger average degeneration degree of the multifidus were independent risk factors for PI-LL mismatch.

## Introduction

Adult degenerative scoliosis (ADS) is a three-dimensional deformity in skeletally mature individuals, defined as a coronal deviation of greater than 10° (1). It is a common condition and often causes significant low back pain (LBP) and disability in adults (2). It has been reported that the incidence rate of ADS is up to 68% in the older population (3). Given its prevalence in the expanding portion of the global population aged older than 65 years, the disorder is of growing interest in the field of healthcare (4). Furthermore, ADS is a complicated deformity and has always been a matter of concern, but its pathogenesis remains unclear.

The paravertebral muscle (PVM) is closely associated with spinal deformities (5–7) and health-related quality of life (HRQOL) (8). The signal intensities and degree of fatty change of the multifidus muscle are higher in people with degenerative lumbar kyphosis than in the healthy population (5). There is a significant imbalance between fatty infiltration and muscle volume in the deep PVM of adolescent idiopathic scoliosis (AIS) (6). Our previous study found that there exists an asymmetric degeneration of the PVM in ADS, and the asymmetric change is more often seen on the concave side (7). In addition, the lumbar PVM fatty infiltration area (FIA) is closely related to LBP and disability in adults (8).

Pelvic incidence (PI) minus the lumbar lordosis (LL) angle (PI-LL) can be used to study the relationship between the pelvic and the lumbar curve, and both of them correlate with PVM degeneration (7, 9, 10). In addition, PI-LL is considered a valuable tool for surgical planning in the treatment of adult patients with spinal deformities (11–13) and correlates with function and disability, both of which are associated with PVM degeneration (5–7). However, the direct relationship between PI-LL and PVM has not been reported to date.

This study aims to reveal the characteristics of the PVM in ADS with PI-LL match or mismatch and identify the risk factors for PI-LL mismatch.

## Materials and methods

### Study design and subjects

This was a retrospective cross-sectional study. Data from patients with ADS who were diagnosed at the Third Hospital of Hebei Medical University during the period from July 2010 to October 2019 were evaluated. The inclusion criteria were as follows: (1) aged ≥50; (2) presence of ADS, defined by a coronal Cobb angle >10°; (3) presence of ADS with no radiculopathy and those who did not receive physical therapy, acupuncture, or brace treatment. The exclusion criteria were as follows: (1) presence of symptomatic spinal stenosis with neurogenic claudication and those who received physical therapy, acupuncture, or brace treatment; (2) a recent history of trauma; (3) a prior diagnosis of scoliosis or other spine deformities; (4) underlying diseases such as diabetes mellitus (DM) and sarcopenia; (5) data integrity. All patients in this study had only symptoms of LBP. The visual analog scale (VAS), symptom duration, and Oswestry disability index (ODI) were used to assess the patients’ clinical symptoms and quality of life.

### Imaging procedures

The radiography system used was a 500 mA Siemens DR System (Siemens Corporation, Germany) with an automatic exposure control system. The detailed parameters are as follows: the electric current was kept at 500 mA and the voltage at 75 kV on the anteroposterior position, and the current was kept at 500 mA and the voltage at 85 kV on the lateral position.

The MRI system was a 1.5 Tesla Imaging System (Siemens Magnetom Symphony, Germany). T1-weighted images (T1WI) and T2-weighted images (T2WI) of sagittal views of the lumbar intervertebral disc were obtained using a spin echo sequence system for T1WI and a fast spin echo sequence system for T2WI. A surface coil was used. The slice width was 4 mm and the interslice gap was 1 mm. The acquisition matrix was 512 × 256. The sequence parameter was repetition time (TR) 482 ms/echo time (TE) 10 ms for T1WI and TR 2,300 ms/TE 99 ms for T2WI.

### Data collection and imaging evaluation

X-ray and MRI were performed for all subjects and the data were recorded in detail.

Radiography was a useful tool to evaluate the bony structural parameters of ADS. The radiography consisted of a standing anteroposterior and lateral radiograph of the entire spine. In standard anteroposterior radiography, the lumbar scoliosis Cobb's angle, apical vertebral level, and curve direction of the main/compensatory curve were measured. A distance between the C7 plumb line (C7PL) and the center sacral vertical line (CSVL) of more than 2 cm represented coronal plane imbalance, and if this distance was less than 2 cm, it denoted coronal plane balance (14). In standard lateral radiography, the measurements were LL, and spinopelvic parameters included PI, pelvic tilt (PT), sacral slope (SS), and PI-LL. A sagittal vertical axis (SVA) <5 cm denoted sagittal plane balance, and if it was >5 cm, it represented sagittal plane imbalance. A total of 13 PI-LL < 10° were defined as the PI-LL match group and PI-LL ≧ 10° as the PI-LL mismatch group (15, 16).

MRI was a valuable tool to assess the changes in the PVM, and T2WI were taken at each L1-SI disc level. The center slice of the multifidus muscle was the primary research subject that included the cross-sectional area (CSA) and the percentage of fat infiltration area (FIA%). The CSA of the multifidus muscle was measured in the following steps. The first step was to set scale pixel/cm, converting pixels into cm measurement units by using the ruler present in the photographic image and converting each image into a grayscale 8-bit image. The second step was to measure the CSA of the multifidus muscle by outlining its region freehand using Image-J software. The measurement of FIA% was based on the above, using a threshold technique. The detailed operation steps were as follows: the value of the threshold was selected by the “default” and “dark background” method automatically and then image/adjust/ threshold/dark background and default/auto. Fat tissue in the 8-bit image was colored red using the threshold technique and the red area was measured as the FIA. FIA% was FIA divided by CSA.

### Statistical analysis

Statistical analysis was conducted using IBM SPSS 20.0 software. Non-parametric tests were used to compare age and the ODI between PI-LL match and mismatch. The *χ*^2^ test was used to compare sex between PI-LL match and mismatch. The *t*-test was used to compare sex, the VAS score, and symptom duration, respectively, between PI-LL match and mismatch. The *t*-test was used to compare the asymmetric degeneration degree and average degeneration degree of the multifidus muscle between the two groups. The Pearson correlation coefficient was used to assess the correlation between the asymmetric and the average degeneration degree of the multifidus muscle and the VAS score and the symptom duration. Univariate and multivariate logistic regression analyses were used to identify the possible risk factors. For all statistical analysis, the level of significance was set at *P* < 0.05.

## Results

### Demographic characteristics

A total of 67 patients who met the above criteria were enrolled in this study, and they included 17 males and 50 females. The mean age was 63.1 ± 7.2 (years) and the mean body mass index (BMI) was 25.4 ± 1.8 (kg/m^2^). The subjects were divided into two groups: PI-LL match group (31 cases) and PI-LL mismatch group (36 cases). The mean age of the PI-LL mismatch group was more likely older than that of the PI-LL match group (65.0 ± 5.5 vs. 61.0 ± 8.4, *P* < 0.05, Table 1), but gender distribution and BMI were not statistically different between the two groups (*P* > 0.05, Table 1).

**Table 1:** Comparison of baseline data between PI-LL match and PI-LL mismatch groups.

Variable	PI-LL match group	PI-LL mismatch group	Statistics	*P*-value
Cases	31	36	—	—
Male/female	9/22	8/28	*χ*^2^ = 0.408	0.523
Age (years)	61.0 ± 8.4	65.0 ± 5.5	*Z* = −2.418	0.016
BMI (kg/m^2^)	25.3 ± 2.0	25.4 ± 1.6	*t* = −0.186	0.853
VAS score	3.52 ± 1.46	4.33 ± 1.60	*t* = −2.169	0.034
Symptom duration (month)	6.58 ± 4.23	10.81 ± 4.83	*t* = −3.779	0.000
ODI	12.97 ± 6.49	21.06 ± 12.58	*Z* = −2.601	0.009

PI-LL, pelvic incidence minus the lumbar lordosis angle; VAS, visual analog scale; ODI, Oswestry disability index.

### VAS score, symptom duration, and ODI in the PI-LL match and mismatch groups

The VAS score in the PI-LL mismatch group was 4.33 ± 1.60, which was higher than that in the PI-LL match group, which was 3.52 ± 1.46 (*P* < 0.05, Table 1). The symptom duration (month) in the PI-LL mismatch group was 10.81 ± 4.83, which was higher than that in the PI-LL match group, which was 6.58 ± 4.23 (*P* < 0.05, Table 1). The ODI in the PI-LL mismatch group was 21.06 ± 12.58, which was higher than that in the PI-LL match group, which was 12.97 ± 6.49 (*P* < 0.05, Table 1).

### The FIA% of the multifidus muscle in the PI-LL match and mismatch groups

In the PI-LL match group, the average FIA% of the multifidus muscle on the convex side was 22.41 ± 8.45 (%), 22.16 ± 8.70 (%), 25.82 ± 9.70 (%), 25.80 ± 7.10 (%), and 29.89 ± 8.61 (%), and on the concave side, it was 31.87 ± 9.05 (%), 35.18 ± 12.32 (%), 32.49 ± 9.89 (%), 33.32 ± 8.72 (%), and 37.77 ± 10.42 (%) at the L1-2, L2-3, L3-4, L4-5, and L5-S1 levels. The average FIA% of the multifidus muscle on the concave side was larger than that on the convex side at each level (*P* < 0.05, Table 2).

**Table 2:** The FIA% of the multifidus muscle on the concave and convex sides in the PI-LL match group.

	Level	Multifidus muscle		*t-*value	*P*-value
		Convex side	Concave side		
FIA%	L1-2	22.41 ± 8.45	31.87 ± 9.05	−4.303	<0.000*
	L2-3	22.16 ± 8.70	35.18 ± 12.32	−8.329	<0.000*
	L3-4	25.82 ± 9.70	32.49 ± 9.89	−5.252	<0.000*
	L4-5	25.80 ± 7.10	33.32 ± 8.72	−6.529	<0.000*
	L5-S1	29.89 ± 8.61	37.77 ± 10.42	−5.846	<0.000*

PI-LL, pelvic incidence minus the lumbar lordosis angle; FIA%, percentage of fat infiltration area.

*Convex side including the convex of the main curve and compensatory curve; significant if *P *< 0.05.

**Figure 1:**
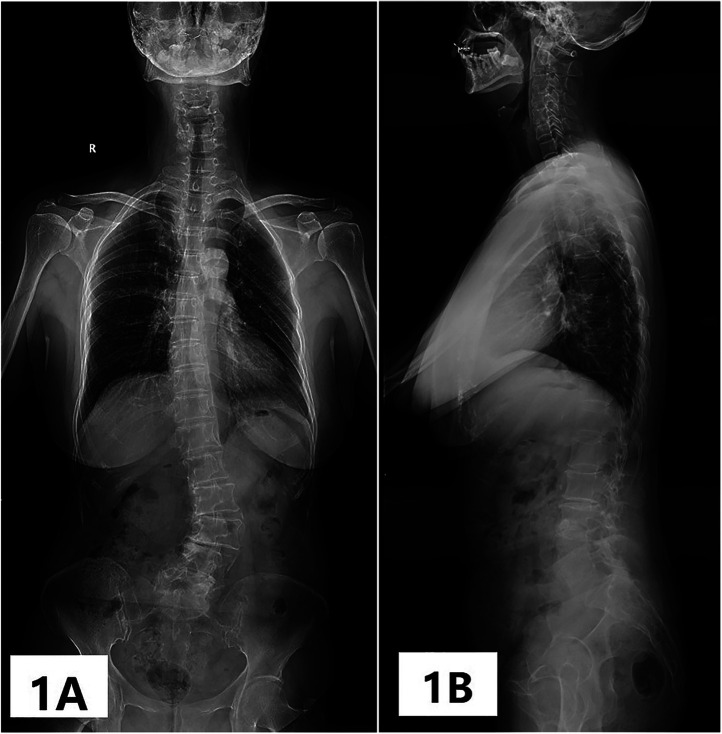
(**A**) Apical vertebrae was L3 vertebrae, the scoliosis Cobb's angle was 40°, and the coronal plane was imbalanced; the main curve was located on the lumbar segment with left scoliosis, and the compensatory curve whose orientation was opposite to the main curve was located on the lumbosacral segment. (**B**) LL = 41°, SS = 36°, PT = 32°, PI = 68°, PI-LL = 27° and the sagittal plane was imbalanced.

**Figure 2:**
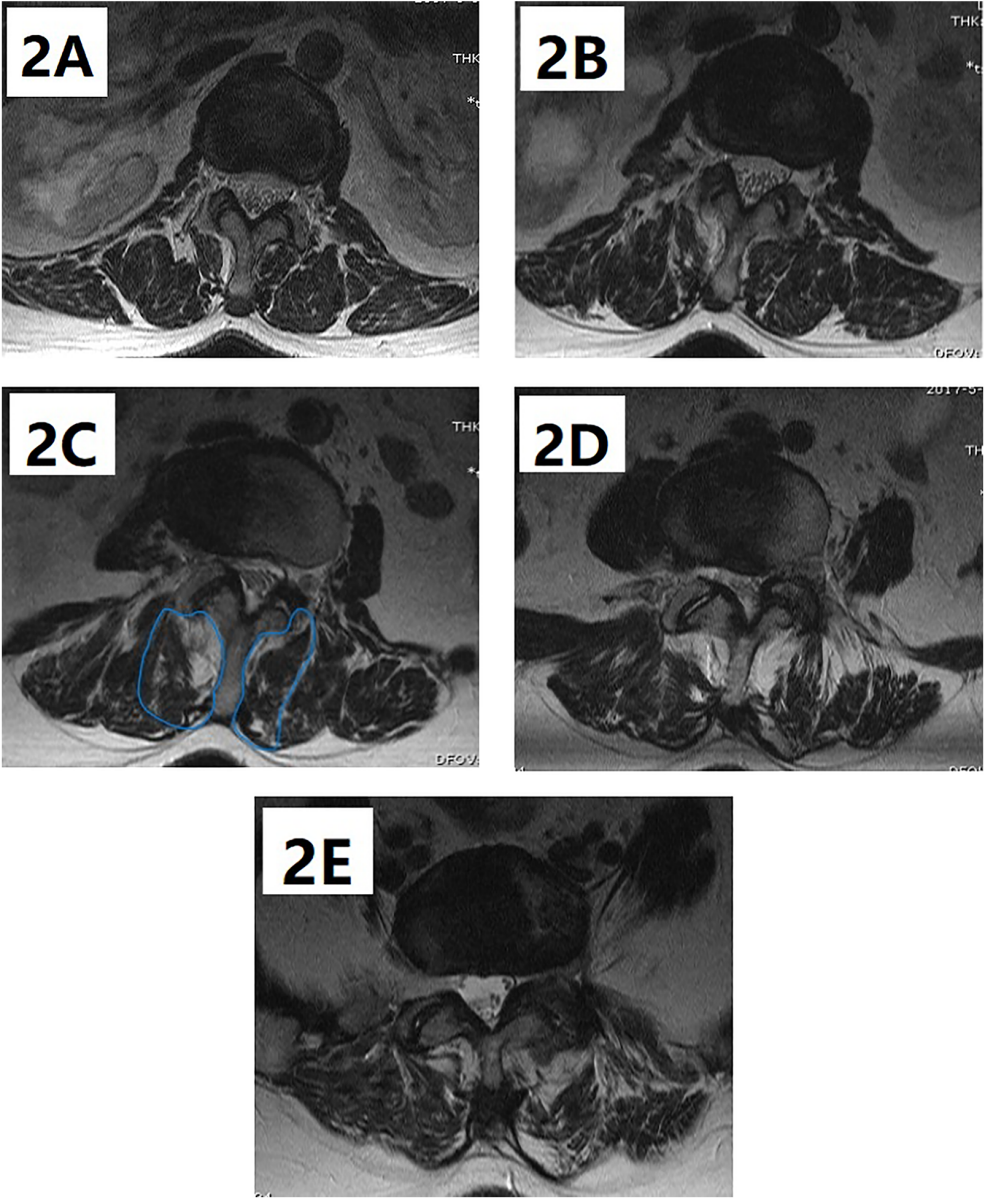
(**A–E**) Fat tissue on the MRI is colored in white; FIA% of the multifidus muscle on the convex side is 27.09%, 14.26%, 30.37%, 33.36%, and 30.69%, and on the concave side, it is 40.67%, 42.77%, 39.25%, 34.16%, and 43.02% at the L1-2, L2-3, L3-4, L4-5, and L5-S1 levels, respectively; the asymmetric degree of the multifidus muscle is 12.82% and the average degree of the multifidus muscle is 33.57%.

**Figure 3:**
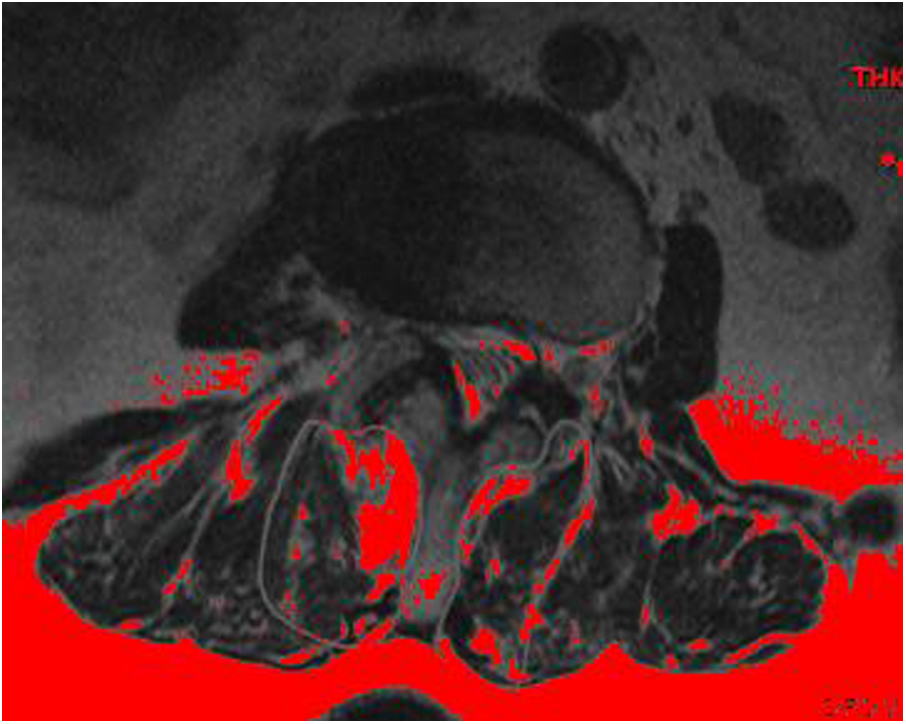
Fat tissue on the MRI is colored in red (a darker background in the red version) using the threshold technique.

In the PI-LL mismatch group, the average FIA% of the multifidus muscle on the convex side was 24.49 ± 7.98 (%), 25.02 ± 9.73 (%), 26.78 ± 9.31 (%), 27.18 ± 7.71 (%), and 31.76 ± 8.64 (%), and on the concave side, it was 36.05 ± 12.14 (%), 38.29 ± 11.85 (%), 35.57 ± 13.19 (%), 36.12 ± 9.60 (%), and 40.95 ± 9.91 (%) at the L1-2, L2-3, L3-4, L4-5, and L5-S1 levels. The average FIA% of the multifidus muscle on the concave side was larger than that on the convex side at each level (*P* < 0.05, Table 3) (Figures 1–3).

**Table 3:** The FIA% of the multifidus muscle on the concave and convex sides in the PI-LL mismatch group.

	Level	Multifidus muscle		*t-*value	*P*-value
		Convex side	Concave side		
FIA%	L1-2	24.49 ± 7.98	36.05 ± 12.14	−6.582	<0.000*
	L2-3	25.02 ± 9.73	38.29 ± 11.85	−6.528	<0.000*
	L3-4	26.78 ± 9.31	35.57 ± 13.19	−4.327	<0.000*
	L4-5	27.18 ± 7.71	36.12 ± 9.60	−5.274	<0.000*
	L5-S1	31.76 ± 8.64	40.95 ± 9.91	−6.614	<0.000*

PI-LL, pelvic incidence minus the lumbar lordosis angle; FIA%, percentage of fat infiltration area.

*Convex side including the convex of the main curve and compensatory curve; significant if *P *< 0.05.

### Differences in the asymmetric degeneration degree and average degeneration degree of the multifidus muscle between PI-LL match and mismatch groups

The asymmetric degeneration degree of the multifidus muscle in the PI-LL match group was 8.91 ± 4.61 (%), and in the PI-LL mismatch group, it was 10.35 ± 5.44 (%), and there was no statistical difference between the two groups (*P* > 0.05, see Table 4). The average degeneration degree of the multifidus muscle in the PI-LL mismatch group was 32.22 ± 6.98 (%), which was higher than that in the PI-LL match group, which was 26.28 ± 6.23 (%) (*P* < 0.05, Table 4).

**Table 4:** Asymmetric and average degeneration degree of the multifidus muscle between PI-LL match and PI-LL mismatch groups.

FIA%	PI-LL match group	PI-LL mismatch group	*t*-value	*P*-value
Asymmetric degeneration degree of the multifidus muscle	8.91 ± 4.61	10.35 ± 5.44	−1.160	0.250
Average degeneration degree of the multifidus muscle	26.28 ± 6.23	32.22 ± 6.98	−3.646	0.001*

PI-LL, pelvic incidence minus the lumbar lordosis angle; FIA%, percentage of fat infiltration area.

*Significant if *P *< 0.05.

### Correlation between asymmetric and average degeneration degree of the multifidus muscle and VAS score, symptom duration, and ODI

In the 67 patients in this study, the asymmetric degree of the multifidus muscle was positively correlated with symptom duration (*r* = 0.403, *P* < 0.05, Table 5). There was no correlation between the asymmetric degree of the multifidus muscle and the VAS score or ODI (*P* > 0.05, Table 5). The average degeneration degree of the multifidus muscle was positively correlated with the VAS score, symptom duration, and ODI, respectively (*r* = 0.515, 0.614, 0.548, *P* < 0.05, Table 5).

**Table 5:** Correlation between asymmetric and average degeneration degree of the multifidus muscle and the VAS score, symptom duration, and ODI.

*r-*value	Asymmetric degeneration degree of the multifidus muscle	Average degeneration degree of the multifidus muscle
VAS score	0.125	0.515*
Symptom duration	0.403*	0.614*
ODI	0.143	0.548*

PI-LL, pelvic incidence minus the lumbar lordosis angle; VAS, visual analog scale; ODI, Oswestry disability index.

*Significant if *P *< 0.05.

### Risk factors associated with PI-LL mismatch

The univariate logistic regression analysis showed that age, scoliosis Cobb angle, sagittal plane balance, LL, PT, SS, and average degeneration degree of the multifidus muscle were independent risk factors for ADS with PI-LL mismatch (*P* < 0.05, Table 6). The binary multivariate logistic regression analysis revealed that sagittal plane balance, LL, PT, and average degeneration degree of the multifidus muscle were risk factors for ADS with PI-LL mismatch (OR: 15.447, 95% CI: 1.274–187.269, *P* = 0.032; OR: 0.001, 95% CI: 0.000–0.099, *P* = 0.004; OR: 107.540, 95% CI: 5.195–2,225.975, *P* = 0.002; OR: 52.531, 95% CI: 1.797–1,535.551, *P* = 0.021, Table 7).

**Table 6:** Univariate logistic regression analysis of significantly different variables for the factors associated with PI-LL mismatch.

	B-value	SE	Wald	*P*-value	Exp (B)	Exp (B), 95% CI
Age	0.871	0.413	4.448	0.035*	2.390	1.064–5.372
Scoliosis Cobb angle	0.608	0.240	6.419	0.011*	1.836	1.147–2.938
Sagittal plane balance	0.153	0.511	5.091	0.024	3.167	1.164–8.619
LL	−4.297	1.085	15.700	0.000*	0.014	0.02–0.114
PT	3.881	1.078	12.971	0.000*	48.462	5.864–400.483
SS	−1.188	0.538	4.871	0.027*	0.305	0.106–0.875
Average degeneration degree of the multifidus muscle	1.381	0.449	9.467	0.002*	3.977	1.651–9.583

SE, standard error; PI, pelvic incidence; LL, lumbar lordosis; SS, sacral slope.

*Significant if *P *< 0.05.

**Table 7:** Multivariate logistic regression analysis of significantly different variables for the factors associated with PI-LL mismatch.

	B-value	SE	Wald	*P*-value	Exp (B)	Exp (B) 95% CI
Sagittal plane balance	2.737	1.273	4.624	0.032*	15.447	1.274–187.269
LL	−7.119	2.455	8.411	0.004*	0.001	0.000–0.099
PT	4.678	1.546	9.155	0.002*	107.540	5.195–2,225.975
Average degeneration degree of the multifidus muscle	3.961	1.722	5.292	0.021*	52.531	1.797–1,535.551

SE, standard error; PI, pelvic incidence; LL, lumbar lordosis.

*Significant if *P *< 0.05.

## Discussion

The multifidus muscle, the most medial PVM, plays a key role in lumbar stability, and is often regarded as the subject of primary research for its sensitivity to pathologic change. In patients with unilateral lumbar disc herniation, there exist ipsilateral abnormal changes in the multifidus muscle (17). In addition, our team found that there existed asymmetric PVM changes in ADS and its asymmetric degree was associated with bony structural parameters such as apical vertebral rotation, lateral vertebral translation, LL, and lumbar scoliosis Cobb's angle (7). For further study, we investigated the relationship between multifidus and PI-LL, and we found that the latter was a novel spinopelvic parameter for surgical planning in the treatment of adult patients with spinal deformities.

Spinopelvic parameters form a part of the most important bony structural parameters and are closely related to disability and quality of life (8, 11, 18, 19). PI-LL mismatch is directly related to poor q;’lpuality of life in ASD patients (11). A higher PI-LL leads to worse LBP and PI-LL mismatch is correlated to postoperative residual symptoms (20). In this study, the VAS score, pain duration, and ODI of patients in the PI-LL mismatch group were found to be higher than in the PI-LL match group, and the results were consistent with those of previous studies. It is important to study PI-LL for understanding the clinical symptoms, progression, and surgical treatment of ADS. The purpose of this study was to reveal the characteristics of the PVM in ADS with PI-LL match or mismatch and identify the risk factors for PI-LL mismatch.

In the PI-LL match and mismatch groups, the FIA% of the multifidus muscle on the concave side was significantly larger than that on the convex side, and the characteristics of the PVM were similar. These results were consistent with those of previous studies on the correlation between PVM and scoliosis (7, 21–23). The FIA was greater on the concave side than on the convex side in AIS, and the multifidus muscle on the convex side was smaller than on the concave side at the apex in idiopathic scoliosis (IS) (21, 22). In ADS, the FIA of the multifidus muscle on the concave side was also significantly higher than on the convex side at the apical vertebral level (23). In our previous study, there existed asymmetric PVM changes in ADS and FIA% on the concave side was higher than on the convex side (7). The above results were consistent with those of this study and revealed that the pattern of PVM change was similar no matter what type of scoliosis it was. Different from former studies, we further divided ADS patients into two groups and found that the characteristics of PVM asymmetric change were the same and were not related to PI-LL match or mismatch. Therefore, it was considered that the asymmetric change resulted from the mechanical asymmetry of the spine in patients with scoliosis and that sagittal parameters had less effect on this asymmetry.

Between the PI-LL match group and the mismatch group, there was no statistical difference in asymmetric degeneration of the multifidus muscle. PI was an anatomical parameter that was relatively fixed and reflected spinopelvic morphotypes (13). LL could reveal the lumbar sagittal plane condition and could be influenced by factors such as age, sex, and disc degeneration (24). Based on this thesis, we considered that LL was a key factor influencing the value of PI-LL. In our previous study, we found that the asymmetric degree was negatively correlated with LL, but the correlation was weak (7). A possible reason was that there existed a negative correlation between LL and scoliosis Cobb's angle and the latter directly influenced the PVM change (7, 25–27). When the LL increased by 10°, the lumbar scoliosis Cobb's angle reduced at least by 5° (25). In this study, a tendency towards increase in PI-LL mismatch could be discerned, although the asymmetry degree was not statistically different between the two groups. Moreover, the asymmetric degree of the multifidus muscle was positively correlated with pain duration, but there was no correlation between the asymmetric degree of the multifidus muscle, VAS score and ODI. We considered that pain could lead to an abnormal posture. The longer the pain duration, the greater asymmetric pressure on the PVM and, finally, the asymmetric degree of the multifidus muscle is aggravated.

In the PI-LL mismatch group, the average degeneration degree of the multifidus muscle was higher than in the PI-LL match group, and the VAS score, pain duration, and ODI were more severe than in the latter group. This result was consistent with the trend of the average degeneration degree of the multifidus muscle. Furthermore, we found that the average degeneration degree of the multifidus muscle was positively correlated with the VAS score, symptom duration, and ODI, respectively. It could be inferred that the possible mechanism was that PI-LL mismatch could aggravate the degeneration of the PVM, and the latter was an important source of pain (8, 28, 29), disability (28), and lumbar instability (30, 31). Wu et al. reported that the VAS score and ODI were significantly positively correlated with the tone and stiffness of the PVM on the painful side (32). Protopsaltis concluded that PI-LL correlated with the ODI and Short Form-36 physical component score scoliosis research society (SRS) correlated with all (33). Skeletal malalignment might result in a greater recruitment in muscular effort and a greater energy expenditure to maintain the erect posture as well as the use of compensatory mechanisms (13). Several studies supported the idea that the appropriate sagittal alignment determined the outcome of spinal deformity surgery and that it is a better determinant of HRQOL and pain in patients (13, 34, 35). A better correction of PI-LL mismatch could result in good clinical outcomes in patients with degenerative flat back deformity and ASD (36). In this study, we clarified the importance of PI-LL and the possible mechanism of pain and disability resulting from PI-LL mismatch and inferred that PI-LL mismatch could aggravate PVM degeneration which was possibly associated with pain and disability in ADS patients.

The present study showed that sagittal plane imbalance, decreased LL, higher PT, and larger average degeneration degree of the multifidus muscle were the independent risk factors for ADS with PI-LL mismatch. There existed a chain of interconnected parameters including SVA, PT, SS, and LL (37, 38). ASD could perturb regional alignment, which might result in a chain of modification along the standing axis. In severe ADS, the consequences were decreased LL, larger PT, and SVA, leading to a “spinopelvic mismatch” radiographically and a resultant loss of function and disability (13). The results of this study were consistent with those of previous studies (13, 15, 16, 37, 38). In addition, the degenerative PVM was less effective in maintaining spinal stability and restoring sagittal plane alignment. We first confirmed that a larger average degeneration degree of the multifidus muscle was an important risk factor for PI-LL mismatch. Lee et al. supported the idea that volume loss of lumbar PVM and ectopic FIA into the PVM may cause spinopelvic deformity (39). Menezes-Reis reported that there was significant correlation between spinopelvic parameters and lumbar PVM volumes (40).

There are also limitations in this study. First, the number of patients with ADS was relatively small. Second, there is a lack of studies on CT, electromyography (EMG), histochemistry, cytology, and pathology to support the findings. In the future, we need to collect more imaging data of patients with ADS through multicenter recruitment and employ more methods of investigation.

## Conclusions

The PVM on the concave side was larger than on the convex side in ADS irrespective of PI-LL was matched or mismatched. PI-LL mismatch could aggravate this abnormal change, which was an important reason for pain and disability in ADS patients. Sagittal plane imbalance, decreased LL, higher PT, and larger average degeneration degree of the multifidus muscle were independent risk factors for PI-LL mismatch. Strengthening of the PVM may be beneficial in preventing PI-LL mismatch in ADS patients.

## Data Availability

The raw data supporting the conclusions of this article will be made available by the authors, without undue reservation.
