# Supplementary figures and images for: Characteristics of the paravertebral muscle in adult degenerative scoliosis with PI-LL match or mismatch and risk factors for PI-LL mismatch
Source: Front Surg. 2023 Mar 29;10:1111024. doi: 10.3389/fsurg.2023.1111024 (PMC10090284; doi:10.3389/fsurg.2023.1111024)

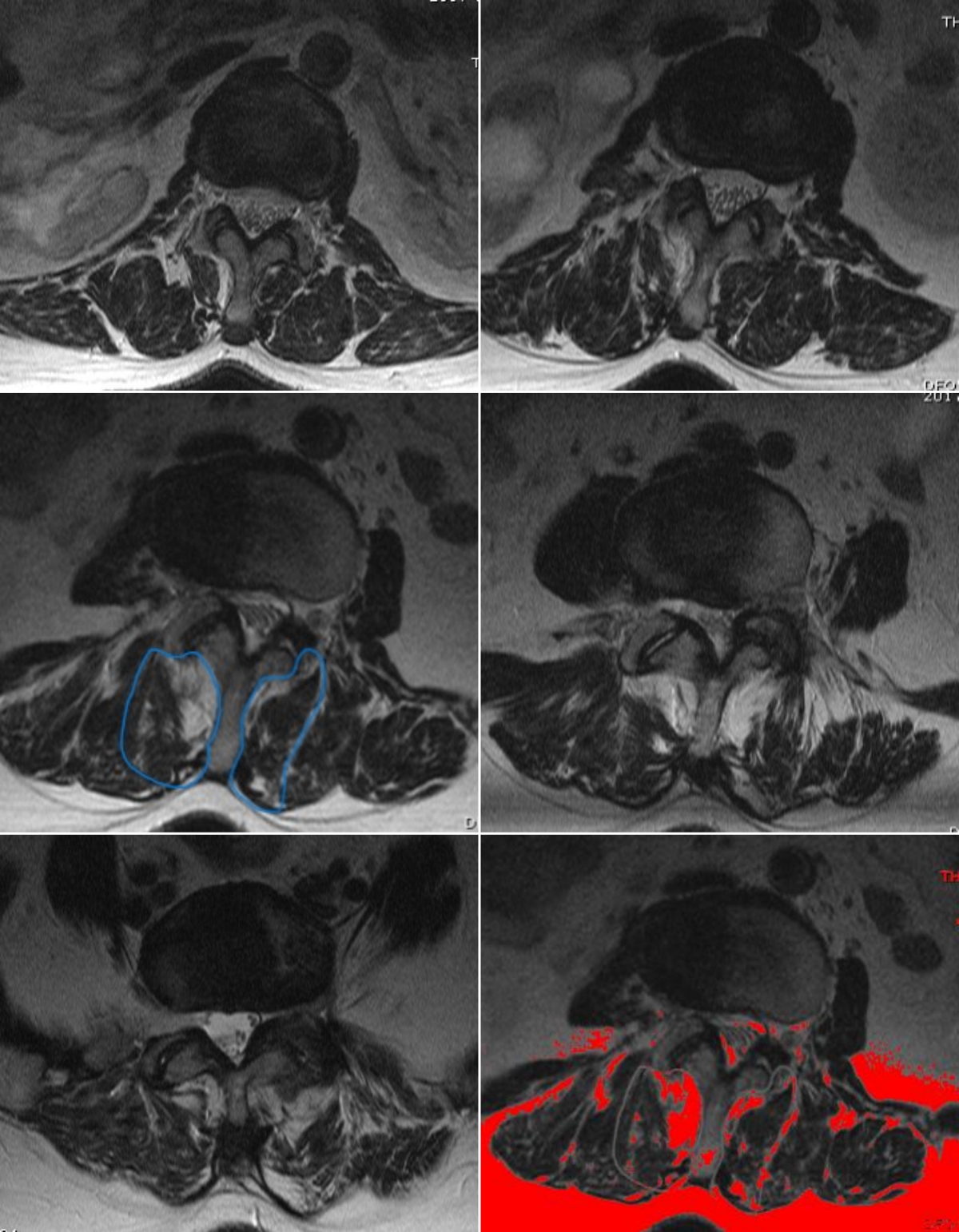

Supplement: Supplementary file 1 [file Image1.jpeg]
